# Comparison of classic and novel human astrovirus MLB and VA seroprevalence in HIV and non-HIV cohorts in South China demonstrates high seroreactivity to classic human astrovirus, which is associated with HIV infection

**DOI:** 10.1128/spectrum.00836-25

**Published:** 2025-06-18

**Authors:** Jianhao Wang, Binyi Liu, Sisi Liu, Yawen Sun, Ruiying He, Yun Lan, Linna Liu, Hongbing Jiang

**Affiliations:** 1 School of Public Health (Shenzhen), Sun Yat-sen University Shenzhen Campus 582261 , Shenzhen, Guangdong, China; 2 Shenzhen Key Laboratory of Pathogenic Microbes and Biosafety, Shenzhen, Guangdong, China; 3 Institute of Infectious Diseases, Guangzhou Eighth People’s Hospital, Guangzhou Medical University, Guangzhou, Guangdong, China; Wright State University, Dayton, Ohio, USA

**Keywords:** human astrovirus, seroepidemiology, HIV cohort

## Abstract

Human astroviruses (HAstVs), including classic and novel clades (MLB, VA), are enteric pathogens with increasing clinical significance, particularly in immunocompromised populations. This cross-sectional study investigated HAstV seroprevalence and antibody response profiles in 197 individuals (101 HIV-positive and 96 HIV-negative) from Guangdong Province, South China. Recombinant HAstV1, MLB2, and VA1 spike proteins were expressed in *Escherichia coli*, structurally validated using AlphaFold2, and used in enzyme-linked immunosorbent assays-based serology with minimal cross-reactivity confirmed in murine models. Cohort study showed that HIV-positive individuals exhibited significantly higher HAstV1 seroreactivity (median OD450: 0.93 vs 0.64, *P* = 0.047) and an ordinal logistic regression revealed greater odds of stronger antibody responses to HAstV1 (adjusted odds ratio [aOR] = 1.91, *P* = 0.027). In contrast, VA1 seroprevalence was lower in HIV-positive participants (median OD450: 0.73 vs 1.02, *P* = 0.026), with reduced odds of high reactivity (aOR = 0.61, *P* = 0.089). In addition, VA1 demonstrated the highest overall seroprevalence (78.68%), followed by MLB2 (77.16%) and HAstV1 (75.13%), and significant associations between co-exposure to MLB2 and VA1 were observed by regression analysis (aOR = 1.60, *P* = 0.010). Together, these findings highlight distinct HIV-associated seroreactivity shifts in HAstV immunity, underscoring the regional importance of classic HAstV1 and the need for clade-specific surveillance in high-risk populations.

**IMPORTANCE:**

Human astroviruses (HAstVs) are increasingly implicated in severe systemic and neurological infections, yet their seroepidemiology in immunocompromised populations remains poorly characterized. This study provides critical insights into the divergent immune responses to classic and novel HAstV clades among people living with HIV (PLWH) in southern China. We demonstrate that PLWH displayed heightened antibody responses to classic HAstV1 but reduced reactivity to MLB2, suggesting HIV-driven immune dysregulation may selectively boost infection with classic HAstV strains. These findings highlight the need for targeted surveillance and improved diagnostics for HAstV infections in high-risk populations.

## INTRODUCTION

Since their first identification in 1975, human astroviruses (HAstVs) remain important yet underrecognized causes of gastroenteritis and have been increasingly implicated in systemic and extraintestinal infections, particularly in immunocompromised individuals (1). As non-enveloped, single-stranded RNA viruses, HAstVs primarily target the gastrointestinal tract, causing diarrheal outbreaks in vulnerable populations, including infants, immunocompromised individuals, and the elderly (2–4). The HAstV genome is approximately 7 kb and consists of four open reading frames (ORF): ORF1a and ORF1b, which encode non-structural proteins involved in replication (5); ORFX, a recently identified conserved overlapping frame, that encodes the protein XP, which functions as a viroporin in virus assembly and release (6); and ORF2, which encodes the capsid precursor protein that undergoes proteolytic processing for virus structural assembly and is the main target for host antibodies (7, 8).

With the emergence of next-generation sequencing technology, novel clades of HAstVs have been discovered in the past decades. For example, the Melbourne (MLB) (9–13) and Virginia/Human-Mink-Ovine-like (VA/HMO) (10, 12, 14–18) genotypes were discovered in stool samples from children with gastroenteritis at different locations across the world since 2008. Since then, HAstV consists of three clades including the classic HAstV (HAstV1-8), the novel HAstV MLB (MLB1-3), and VA/HMO (VA1-6). The classic HAstV1-8 has long been associated with childhood diarrhea, while MLB (MLB1-3) and VA/HMO (VA1-6) have broadened the spectrum of astrovirus-related diseases (19). Recently, both classic and novel HAstVs have been linked to central nervous system (CNS) infections and severe disease in immunosuppressed populations. Several case report studies have documented MLB1 (20), MLB2 (21, 22), VA1 (23–26), classic HAstV1 (27), and HAstV4 (28) in cases of encephalitis, particularly in patients with immunodeficiencies, including organ transplantation and primary immunodeficiency disorders. These findings suggest that HAstVs may cause opportunistic neurotropic infections in susceptible individuals. However, the seroprevalence and epidemiological characteristics of these emerging and classic HAstV genotypes remain poorly understood, particularly in high-risk groups such as people living with HIV (PLWH).

HIV infection is associated with immune dysfunction, which increases susceptibility to enteric and systemic viral infections (29, 30). Previous studies have demonstrated higher seroprevalence rates of various enteric pathogens in HIV-positive individuals (31), raising concerns about the potential burden and clinical significance of HAstVs in this population. Furthermore, given the increasing recognition of astroviruses in neurological diseases, it is critical to evaluate whether HIV-associated immunosuppression facilitates CNS invasion by astroviruses. Thus, interrogating the seroprevalence of classic and novel HAstVs in both HIV-positive and HIV-negative cohorts is crucial for assessing their public health impact and guiding future diagnostic and therapeutic interventions.

In this study, we conducted a comparative seroepidemiological analysis of classic HAstV1, novel HAstV MLB2, and VA1 in HIV-positive and HIV-negative cohorts in southern China. Using enzyme-linked immunosorbent assays (ELISA) to screen for astrovirus seropositivity, we determined the prevalence of these viruses and assessed differences in infection rates between HIV-positive and HIV-negative groups. Our findings highlight the highest seroreactivity to classic HAstV1 in the HIV-positive cohort, providing insights into the epidemiological dynamics of HAstV infections in this region and emphasizing the need for increased surveillance and clinical awareness of astrovirus-associated diseases, such as CNS infections, in immunosuppressed populations.

## RESULTS

### Expression and purification of the recombinant spikes of classic and novel human astroviruses

**Fig 1:**
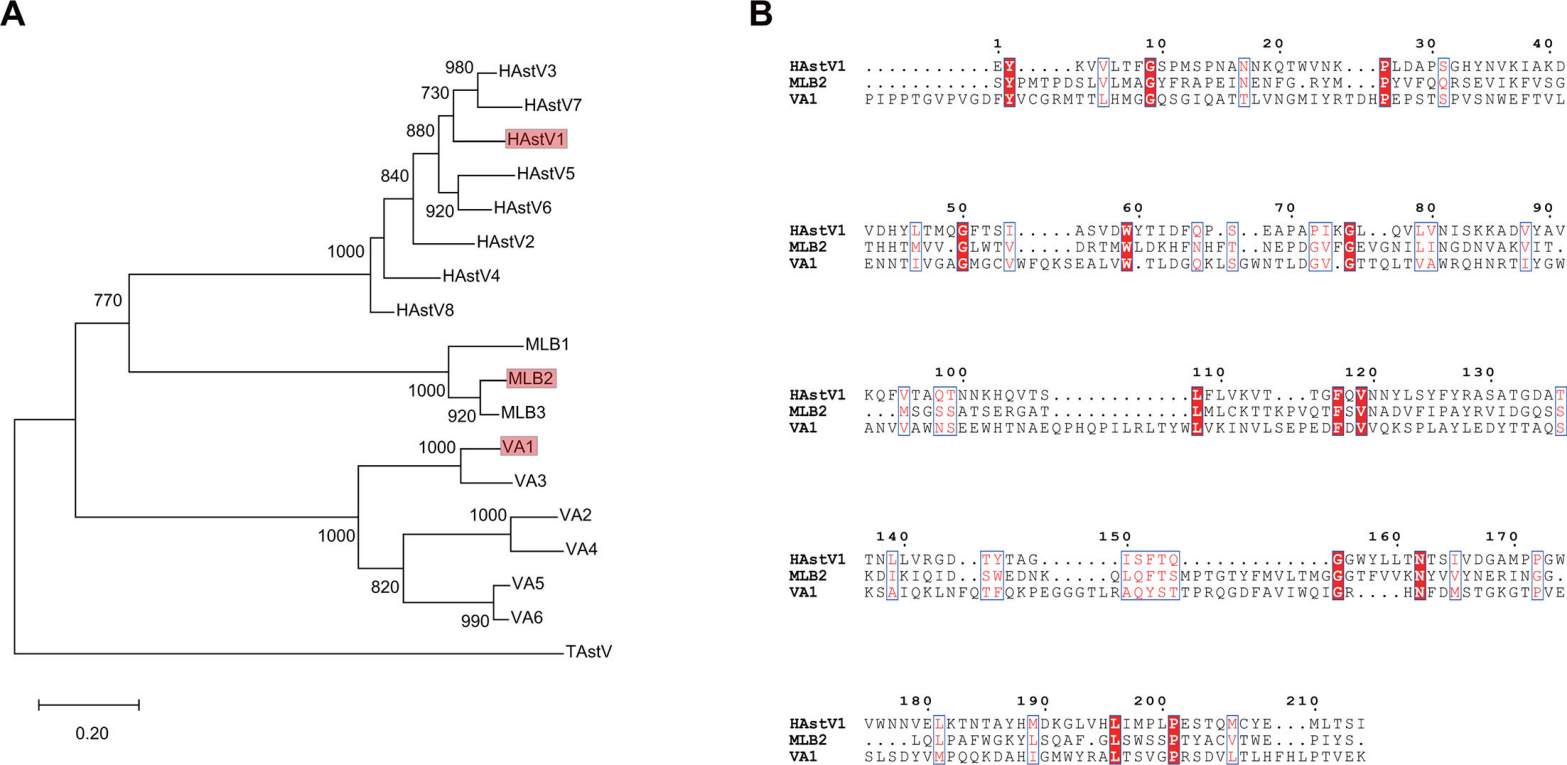
Phylogenetic tree and sequence alignment. (**A**) A phylogenetic tree was constructed by aligning classic and novel HAstV ORF2 amino acid sequences using the maximum likelihood method with 1,000 bootstrap replicates. Turkey astrovirus (TAstV) was chosen as an outgroup. Bootstrap values above 700 were shown. The branch lengths are measured in the number of substitutions per site. (**B**) Alignment of amino acid sequences of HAstV1, MLB2, and VA1 spike protein using MUSCLE and visualized with ESPript 3.0.

**Fig 2:**
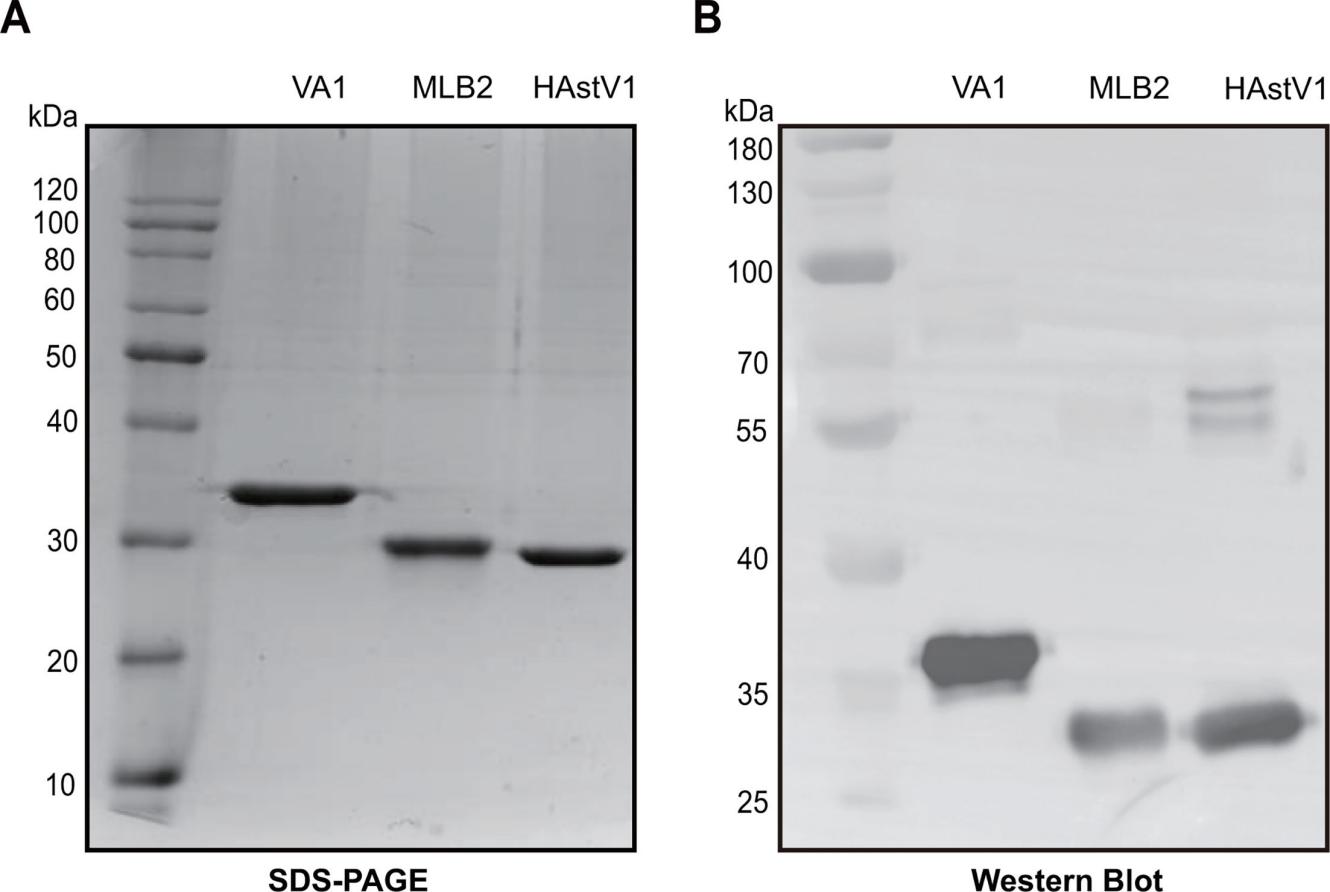
Recombinant spike proteins expressed in *E. coli*. (**A**) Coomassie blue staining of an SDS-PAGE showing His-tagged HAstV1, MLB2, and VA1 spike protein. (**B**) Western blot of recombinant His-tagged spike proteins.

In order to assess the seroepidemiology of HAstVs, we chose to express the spike domain, which is the dominant antigenic region of the astrovirus capsid shell (32–34). The classic HAstV1, novel HAstV MLB2, and VA1 spike were chosen to express in *Escherichia coli* as they represent each HAstV clade according to our phylogenetic analysis (Fig. 1A). HAstV1, MLB2, and VA1 spike proteins are rather divergent according to their sequence alignment (Fig. 1B) and were each expressed with a six histidine tag at both N- and C-terminus and affinity purified with Nickel–Nitrilotriacetic Acid chromatography columns. SDS-PAGE and Coomassie blue staining were used to assess the identity and quality of these proteins. Results showed that all three recombinant spike proteins migrated as a pure single band to their corresponding nominal molecular weights (ca. 28.2 kDa for HAstV1, ca. 29.6 kDa for MLB2, and ca. 35.1 kDa for VA1; Fig. 2A). Additionally, western blot using antibodies against the 6 × His tag developed specific bands of their expected sizes (Fig. 2B).

**Fig 3:**
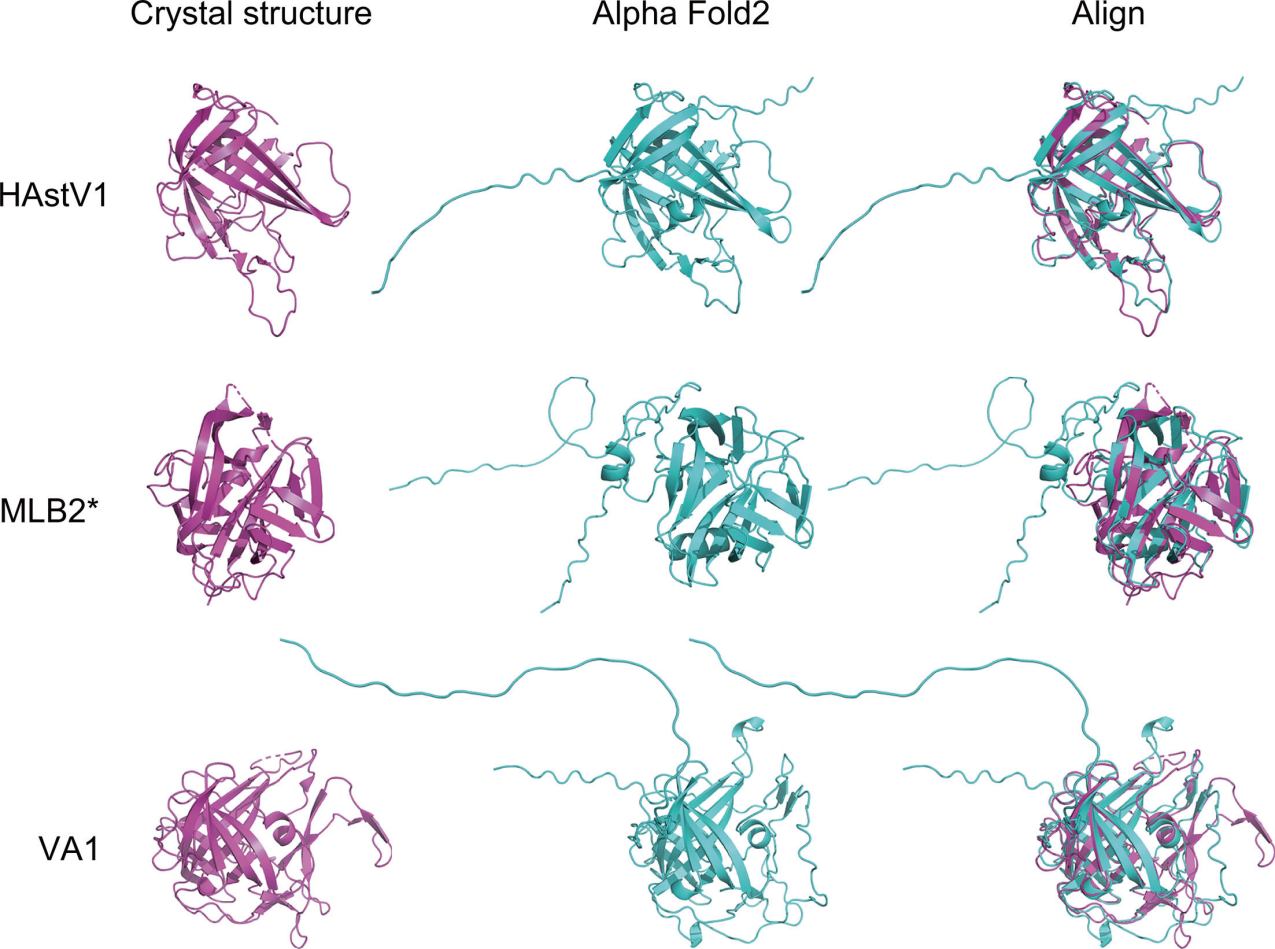
Structural alignment of HAstV spike crystal structures with AlphaFold2-predicted structures. AlphaFold2-predicted HAstV1 and VA1 recombinant spike models were aligned with their corresponding crystal structures (HAstV1 PDB ID: 5EWO and VA1 PDB ID: 8UFO). AlphaFold2-predicted recombinant MLB2 spike structure was aligned with the homologous MLB1 crystal structure (PDB: 7UZT). Crystal structures are shown in magenta and AlphaFold2 models in cyan; * indicates alignment with the homologous crystal structure.

To further reveal that the designed spikes are well folded after expression, we used *in silico* AlphaFold2 structure prediction and structure alignment. Structural alignment of the spike domains from the experimentally determined HAstV1 and VA1 capsid crystal structures with the AlphaFold2 predicted expression constructs using PyMOL software revealed a root mean square deviation (RMSD) of 0.694 Å across 173 residue pairs of HAstV1 and an RMSD of 0.918 Å across 181 residue pairs of VA1, suggesting a relatively accurate match in the beta-strand and alpha-helical regions (Fig. 3) (35, 36). In addition, the predicted model of MLB2 was compared with the crystal structure of MLB1 because of the lack of experimentally determined data for MLB2 spike, which led to a more structurally divergent alignment (RMSD ~2.075 Å across 143 residue pairs) (37). As such, our results indicate that the key structures of the recombinant proteins are relatively stable to establish an ELISA. Collectively, our *in silico* analysis and *in vitro* protein expression of the three astrovirus spikes demonstrated that they are quality antigens for ELISA serology studies.

### Analysis of cross-reactivity among HAstV1, MLB2, and VA1 spikes in experimental serum samples

**Fig 4:**
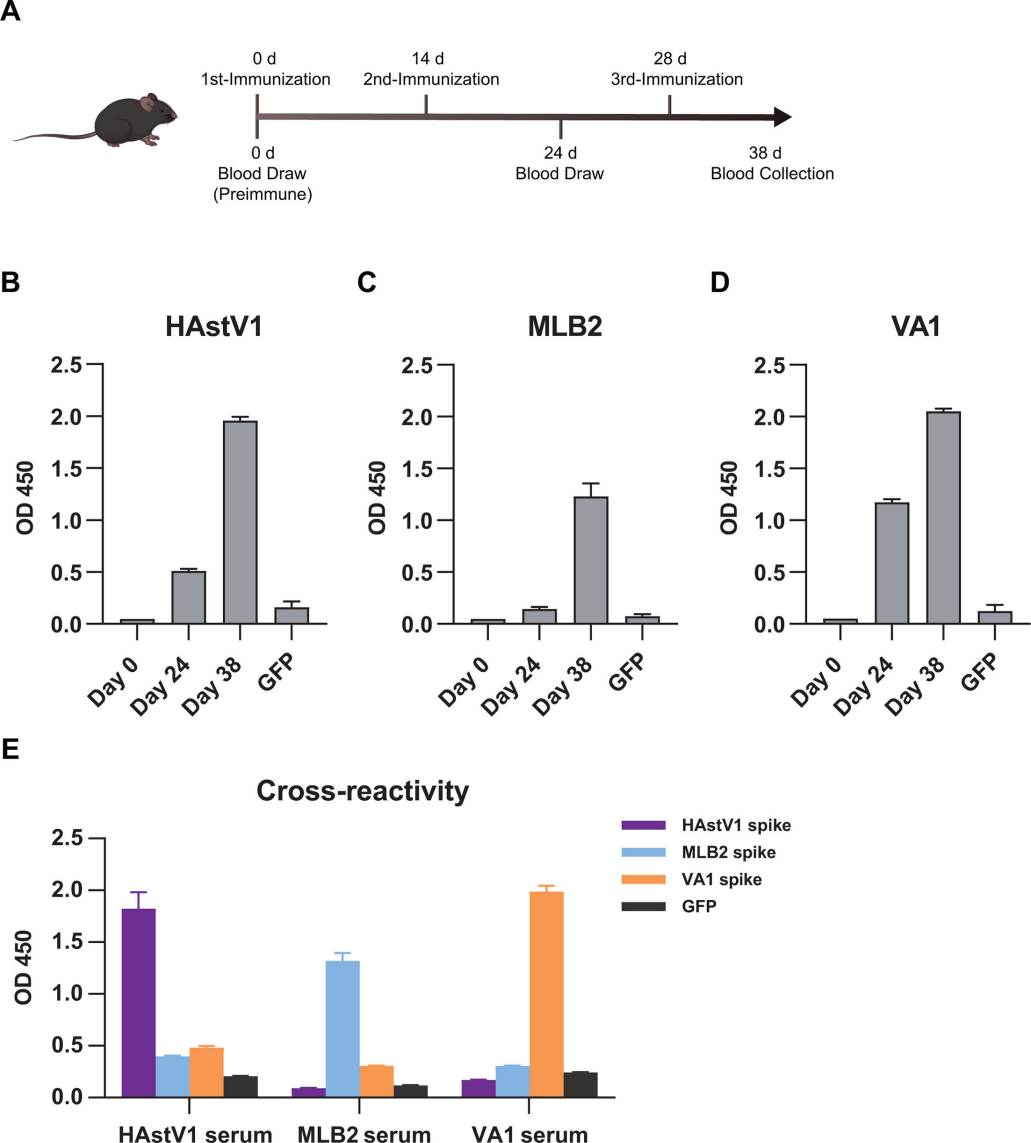
Induction of spike-specific antibodies in mouse and antigenic cross-reaction among HAstV spikes. (**A**) Flow chart of mouse immunization with HAstV spikes. (B–D) ELISA test on spike-immunized mouse serum with corresponding spike antigens on different days. GFP protein served as negative controls. HAstV1, MLB2, and VA1 spike-specific antibody response tested. (**E**) ELISA was performed for cross-reactivity among spike-immunized mouse serum samples.

In order to analyze the cross-reactivity among HAstV1, MLB2, and VA1 spike antigens, we immunized B6/J mice by intramuscular injection using a nano-adjuvant regimen. Preimmunized mouse sera were collected on day 0, and immunized mouse sera were collected on days 24 and 38 post-first immunization (Fig. 4A). The sera of mice were analyzed by indirect ELISA, with the optical density at 450 nm (OD450) measured to evaluate spike antigen-specific IgG responses. Results showed that spike immunization with HAstV1, MLB2, or VA1 all induced specific antibody responses on days 24 and 38 post-immunization as revealed by indirect ELISA (Fig. 4B through D). For the specific antibody induction test, sera from HAstV1-, MLB2-, or VA1-immunized mice exhibited robust reactivity against their own immunized antigens (on day 38, HAstV1 group: mean OD450 = 1.96 ± 0.06; MLB2 group: 1.23 ± 0.22; VA1 group: 2.05 ± 0.05) compared to the Green Fluorescent Protein control coated wells (all *P* < 0.05), indicating spike-specific mouse serum antibodies generated.

For inter-antigenic group cross-reactivity assessment, each spike antigen-coated plate was assayed with the sera of all three antigen-immunized mice by ELISA (Fig. 4E). HAstV1-immunized sera showed specific reactivity to HAstV1 (OD450 = 1.82 ± 0.27) but with negligible reactivity against VA1 (OD450 = 0.48 ± 0.03) or MLB2 (OD450 = 0.40 ± 0.01). Similarly, MLB2-immunized sera showed specific reactivity to MLB2 (OD450 = 1.32 ± 0.13) but only background-level reactivity against HAstV1 (OD450 = 0.10 ± 0.01) and VA1 (OD450 = 0.31 ± 0.01); and VA1-immunized sera displayed specific reactivity to VA1 (OD450 = 1.99 ± 0.09) but no cross-reactivity with HAstV1 (OD450 = 0.17 ± 0.01) or MLB2 (OD450 = 0.42 ± 0.02). The specific mouse sera reactivity and absence of inter-group cross-reactivity confirm the high antigenic specificity of the expressed HAstV1, MLB2, and VA1 spike proteins. Collectively, these findings validated the suitability of spikes as specific capture antigens for subsequent seroepidemiological studies using human clinical samples.

### Seroprevalence and seroreactivity patterns of HAstV1, MLB2, and VA1 in HIV and non-HIV cohorts

**Fig 5:**
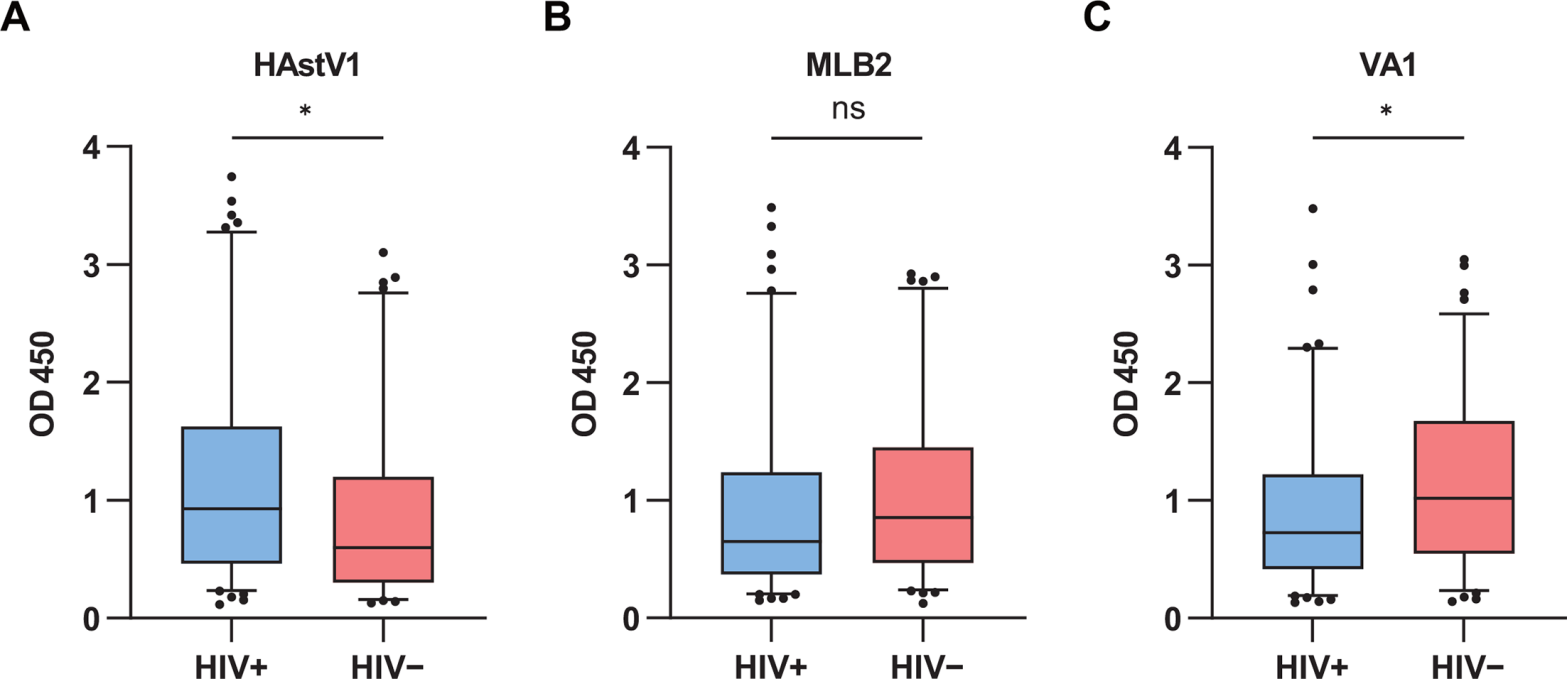
Seroreactivity profiles to HAstV1, MLB2, and VA1 spikes in HIV-positive (*n* = 101) and HIV-negative (*n* = 96) cohorts. Box plots comparing serum OD450 values against HAstV1, MLB2, and VA1 spikes between HIV-positive and HIV-negative cohorts. Horizontal lines denote medians, boxes represent IQR, and whiskers indicate data ranges. Statistical significance was assessed using Mann-Whitney *U* tests. (*, *P* < 0.05).

In order to assay the seroprevalence of HAstV1, MLB2, and VA1 in HIV and non-HIV cohorts in southern China, 197 serum samples from Guangdong Province, South China (HIV+: *n* = 101; HIV−: *n* = 96), were collected and analyzed using indirect ELISA to determine OD450 values against HAstV1, MLB2, and VA1 spike antigens. The purified exotic GFP protein was chosen for background reactivity detection in all serum samples. Box plot visualization and Mann-Whitney *U* tests revealed distinct seroreactivity patterns between HIV+ and HIV− groups. HIV+ individuals exhibited significantly higher OD450 values against HAstV1 (median interquartile range [IQR]: 0.93 [0.47–1.62] vs 0.60 [0.30–1.20], *U* = 3841, *P* = 0.012), indicating higher seroreactivity of HAstV1 in HIV+ than that in the HIV− group. In contrast, VA1 reactivity was slightly lower in the HIV+ cohort (0.73 [0.42–1.21] vs 1.02 [0.42–1.21], *U* = 5741.5, *P* = 0.026), while no significant difference was observed for MLB2 (0.65 [0.38–1.23] vs 0.85 [0.47–1.44], *U* = 5625.5, *P* = 0.052; Fig. 5).

Due to the skewed distribution of OD450 values (Shapiro test *P* < 0.05), the cutoff value was established using the 97.5th percentile (P97.5) of OD450 readings from GFP-coated wells (Table 1). Overall, the total positivity rates were highest for VA1 (78.68%, 95% confidence intervals [CI]: 72.29%–84.18%), followed by MLB2 (77.16%, 95% CI: 70.65%–82.82%) and HAstV1 (75.13%, 95% CI: 68.48%–81.00%). No significant differences in seropositivity were observed across astrovirus genotypes (*χ*² = 0.71, *P* = 0.702). In subgroup analyses stratified by HIV status, significant differences in seropositivity patterns emerged. The HAstV1 seropositivity rate was markedly higher in the HIV+ group (82.18%, 95% CI: 73.30%–89.08%) compared to the HIV− group (67.71%, 95% CI: 57.39%–76.90%; *χ*² = 4.77, *P* = 0.029). However, no significant disparities were observed for MLB2 (HIV+: 72.28% vs HIV−: 82.89%; *χ*² = 2.26, *P* = 0.133) or VA1 (HIV+: 77.23% vs HIV−: 80.21%; *χ*² = 0.11, *P* = 0.737).

**TABLE 1:** Demographic and seroreactivity profiles of the HIV-positive and HIV-negative cohorts^*a*^

Variable	Total	HIV		*P*-value
	(*n* = 197)	+ (*n* = 101)	− (*n* = 96)	
Age, median (IQR)	45 (33–55)	39 (32–52)	50 (37–57)	0.011*
Male, *n* (%, 95% CI)	153 (77.66%, 71.20–83.28%)	93 (92.08%, 84.99–96.52%)	60 (62.50%, 52.03–72.18%)	<0.001***
HAstV1, *n* (%, 95% CI)				
Super strong positive	28 (14.21%, 9.66–19.88%)	17 (16.83%, 10.12–25.58%)	11 (11.46%, 5.86–19.58%)	0.381
Strong positive	32 (16.24%, 11.38–22.15%)	18 (17.82%, 10.92–26.70%)	14 (14.58%, 8.21–23.26%)	0.672
Weak positive	88 (44.67%, 37.60–51.90%)	48 (47.52%, 37.49–57.70%)	40 (41.67%, 31.86–52.18%)	0.494
Total	148 (75.13%, 68.48–81.00%)	83 (82.18%, 73.30–89.08%)	65 (67.71%, 57.39–76.90%)	0.029*
MLB2, *n* (%, 95% CI)				
Super strong positive	19 (9.64%, 5.91–14.65%)	9 (8.91%, 4.16–16.24%)	10 (10.42%, 5.11–18.32%)	0.907
Strong positive	38 (19.29%, 14.03–25.50%)	17 (16.83%, 10.12–25.58%)	21 (21.88%, 14.08–31.47%)	0.474
Weak positive	95 (48.22%, 41.07–55.44%)	47 (46.53%, 36.55–56.73%)	48 (50.00%, 39.62–60.38%)	0.731
Total	152 (77.16%, 70.56–82.82%)	73 (72.28%, 62.48–80.72%)	79 (82.29%, 73.17–89.33%)	0.133
VA1, *n* (%, 95% CI)				
Super strong positive	26 (13.20%, 8.81–18.74%)	10 (9.90%, 4.86–17.46%)	16 (16.67%, 9.84–25.65%)	0.233
Strong positive	34 (17.26%, 12.26–23.27%)	16 (15.84%, 9.33–24.45%)	18 (18.75%, 11.51–28.00%)	0.725
Weak positive	95 (48.22%, 41.07–55.44%)	52 (51.49%, 41.33–61.55%)	43 (44.79%, 34.63–55.29%)	0.425
Total	155 (78.68%, 72.29–84.18%)	78 (77.23%, 67.82–84.98%)	77 (80.21%, 70.38–87.64%)	0.737

^*a*^ *, *P* < 0.05; **, *P* < 0.01, ***, *P* < 0.001.

To further characterize each astrovirus seropositivity and their relation to individual HIV status, ELISA results were stratified into four categories based on OD450 values: negative (OD450 ≤ 1 × cutoff), weak positive (1 × cutoff < OD450 ≤ 3 × cutoff), strong positive (3 × cutoff < OD450 ≤ 5 × cutoff), and super strong positive (OD450 > 5 × cutoff; Table 1). HAstV1 exhibited the highest proportion of super strong positive reactions (14.21%, 95% CI: 9.66%–19.88%), particularly among HIV+ individuals (HIV+: 16.83% [95% CI: 10.12%–25.58%] vs HIV−: 11.46% [95% CI: 5.86%–19.58%]). In contrast, VA1 and MLB2 showed seemingly elevated super strong positive reactivity in the HIV− group (MLB2 HIV−: 10.42% [95% CI: 10.12%–25.58%] vs MLB2 HIV+: 8.91% [95% CI: 4.16%–16.24%]; VA1 HIV−: 16.67% [95% CI: 9.84%–25.65%] vs VA1 HIV+: 9.90% [95% CI: 4.85%–17.46%]), but it was not statistically significant. Weak positive rates dominated across all antigens (HAstV1: 44.67%, 95% CI: 37.60%–51.90%; MLB2: 48.22%, 95% CI: 41.07%–55.44%) without HIV status-associated variations. Despite this refined stratification, no statistically significant differences in graded seropositivity distribution were observed between HIV+ and HIV− groups for any antigen (all *P* > 0.05).

Subsequently, ordinal logistic regression analysis was applied to evaluate the respective predictors of graded seropositivity to HAstV1, MLB2, and VA1. Each ordinal logistic regression model was constructed by including HIV status as the primary predictor, adjusted for age and sex, and simultaneously accounting for co-exposure to the other two viruses (Table 2). Results showed that HIV+ status was associated with 0.91 increased odds of higher HAstV1 response categories (adjusted odds ratio [aOR] = 1.91, 95% CI: 1.08–3.39, *P* = 0.027) but showed no significant associations with MLB2 seropositivity (aOR = 0.72, 95% CI: 0.42–1.35, *P* = 0.344) or VA1 seropositivity (aOR = 0.61, 95% CI: 0.34–1.08, *P* = 0.089). Virus co-exposure analyses revealed that MLB2 OD450 levels were independently associated with elevated VA1 responses (aOR = 1.60, 95% CI: 1.12–2.29, *P* = 0.010), whereas other co-exposure pairs showed no significant associations (all *P* > 0.05). Neither age nor sex demonstrated significant associations across models (all *P* > 0.05). All variance inflation factors (VIFs) were below 5 (VIF < 5), indicating no multicollinearity among the independent variables. The proportional odds assumption was validated for all models through Brant tests (HAstV1: *χ*² = 10.29, *P* = 0.416; MLB2: *χ*² = 2.94, *P* = 0.983; VA1: *χ*² = 10.37, *P* = 0.409), indicating acceptable model calibration.

**TABLE 2:** aOR for seroreactivity profiles based on ordinal logistic regression analyses

Variable	HAstV1 positivity	MLB2 positivity	VA1 positivity
	aOR (95% CI)	aOR (95% CI)	aOR (95% CI)
Age (per year)	0.99 (0.98–1.01)	0.99 (0.98–1.01)	1.00 (0.98–1.01)
Male (vs female)	1.08 (0.57–2.06)	0.71 (0.37–1.38)	1.53 (0.78–3.05)
HIV+ (vs HIV−)	1.91 (1.08–3.39)^*a*^	0.72 (0.42–1.35)	0.61 (0.34–1.08)
Antigen co-exposure			
HAstV1	–^*b*^	1.07 (0.78–1.46)	1.15 (0.83–1.58)
MLB2	1.15 (0.82–1.62)	–	1.60 (1.12–2.29)^*a*^
VA1	1.42 (0.98–2.07)	1.43 (0.98–2.11)	–

^*a*^ *, *P* < 0.05.

^*b*^ ”–”, indicates data not applicable under the experimental conditions.

Collectively, these results highlight distinct HAstV seroreactivity profiles between HIV+ and HIV− cohorts in southern China, with classic HAstV1 showing heightened seroprevalence and antibody reactivity in HIV+ individuals, independent of the demographic variables of the cohorts.

## DISCUSSION

In this study, we conducted a comprehensive seroepidemiological comparison of classic HAstV1, novel HAstV MLB2, and VA1 in HIV-positive and HIV-negative cohorts in southern China. Our findings demonstrate higher seroreactivity to classic HAstV1 in PLWH compared to HIV-negative individuals, while the seroreactivity to novel HAstVs, including MLB2 and VA1, did not show a statistically significant difference between the two cohorts. These results provide valuable insights into the epidemiology of HAstV infections in HIV-positive and HIV-negative populations and highlight the need for further investigation into their clinical implications.

The specificity is critical given the possible cross-reaction between closely related HAstV clades since cross-reactive antibodies could confound seroprevalence and seroreactivity evaluation in clinical samples. Our robust expression and purification of the HAstV1, MLB2, and VA1 spike proteins established a reliable foundation for serological analyses using human samples. The dominant antigenic nature of HAstV spikes was validated by structural alignment, and their minimal cross-reactivity was analyzed through mouse immunization and cross ELIAS assays, demonstrating high reliability for HAstV serological analyses.

The observed high seroreactivity to classic HAstV1 in HIV-positive individuals in our cohorts suggests increased exposure or prolonged viral shedding of HAstV1 due to immune deficiency. HIV-associated immunosuppression is known to increase susceptibility to various enteric viral infections, leading to prolonged viral replication and increased transmission risk. This phenomenon has been observed in other enteric viruses, such as noroviruses and rotaviruses, where immunocompromised individuals exhibit prolonged viral shedding (38). Given that classic HAstVs are highly prevalent and primarily cause self-limiting gastroenteritis, further studies of larger cohorts are needed to determine whether persistent infection or subclinical shedding occurs more frequently in HIV-positive individuals. Interestingly, in our study, novel HAstV-MLB2 and VA1 demonstrated higher overall seroprevalence compared to the classic HAstV1 in the general cohort. While previous studies in Mexico (39), St. Louis, and Peruvian Amazon (40) have similarly reported over 75% seroprevalence rates for VA1, our findings contrast with prior seroprevalence patterns in which HAstV1 typically dominates (41–44). The regional predominance of novel HAstVs may reflect unique transmission dynamics, environmental reservoirs, or immunological naivety to the clades.

The robust positive correlations observed between novel HAstV-MLB2 and VA1 antigen pairs, despite the lack of cross-reactivity in murine models, point to co-exposure rather than cross-priming or antibody-mediated mechanisms as the primary driver. Shared risk factors (e.g., fecal-oral transmission pathways) or concurrent infections likely underlie these associations. Future investigations combining molecular pathogen detection with epidemiological behavioral data are required to elucidate the precise interplay of these exposure-related mechanisms.

We acknowledge several limitations in this study. First, the single-center (urban tertiary hospital) design and cross-sectional sampling limit generalization and preclude causal inferences. Second, the lack of CD4+ T cell counts and other immunological parameters precluded precise assessment of HIV disease progression and host immune status. Third, seropositivity reflects cumulative exposure rather than active infection, necessitating longitudinal studies to delineate infection timelines. Despite these reflections, our findings underscore the high seroprevalence of all three HAstV clades in southern China and reveal HIV-associated shifts in HAstV seroreactivity. The elevated super strong positive rates in HIV+ individuals with classic HAstV1 raise clinical concerns, as severe HAstV outcomes, including encephalitis and disseminated infections, are increasingly reported in immunocompromised patients (25, 45). Routine HAstV screening in diarrhea surveillance programs, particularly for PLWH, could improve diagnostic accuracy and inform therapeutic strategies.

In conclusion, our study defines HAstV seroepidemiology in southern China, highlighting HIV-associated alterations in antibody response profiles. These insights advocate for genotype-specific surveillance and targeted prevention strategies in high-risk populations.

## MATERIALS AND METHODS

### Description of samples and ethics approval

All the serum samples were collected from Guangzhou Eighth People’s Hospital, including 101 from HIV patients and 96 from non-HIV people. The study was approved by the Institutional Review Board of Guangzhou Eighth People’s Hospital, Guangzhou Medical University (Approval No. Ke 202353290). All animal experiments were approved by the Experimental Animal Management and Use Committee of Sun Yat-sen University (Approval Number: SYSU-IACUC-2024-000421).

### Phylogenetic tree and amino acid sequences alignment

All astrovirus ORF2 amino acid sequences were obtained from the National Center for Biotechnology Information (sequence IDs are listed in Table 3). The sequence alignment was completed with the L-INS-i algorithm in Mafft (version 7.505). A phylogenetic tree was constructed by the maximum likelihood method using MEGA (version 11.0) with 1,000 bootstrap replicates based on the Jones-Taylor-Thornton model. Multiple sequence alignment of HAstV1, MLB2, and VA1 spike proteins was performed using MUSCLE in MEGA (version 11.0) and visualized with ESPript 3.0.

**TABLE 3:** Sequence information for constructing phylogenetic tree

Viral strain	Accession no.
HAstV1 Hu/US/2013/CA-RGDS-1071	QKW90824.1
HAstV2 Oxford	AZB52195.1
HAstV3 Oxford	AZB52198.1
HAstV4 Oxford	AZB52201.1
HAstV5 Oxford	AZB52204.1
HAstV6 Oxford	AZB52207.1
HAstV7 Oxford	AZB52210.1
HAstV8 Oxford	AZB52213.1
VA1 C-P5	ASJ26376.1
VA2 VA2/human/Stl/WD0680/2009	ACX83591.2
VA3 VA3/human/Vellore/28054/2005	YP_006905860.1
VA4 VA4/human/Nepal/S5363/2008	YP_006905857.1
VA5 NA46-8	ATW63656.1
VA6 VA6/Sewage/JN/CHN/273/2019	UQK62278.1
MLB1 NAGANO1545	BAU68081.1
MLB2 MLB-YJMGK	AZU90755.1
MLB3 MLB3/human/Vellore/26564/2004	YP 006905854.1
Turkey astrovirus TAstV/CA/00	ABX46569.1

### Structural alignment and RMSD analysis

Structural alignment of the experimentally determined crystal structures (retrieved from the Protein Data Bank, PDB) and AlphaFold2-predicted models (generated in-house for HAstV1, MLB2, and VA1) was performed using PyMOL software (version 3.1.3.1). For alignment, only residues corresponding to the ORF2-encoded spike protein sequences were included to ensure sequence-specific structural comparisons. The PyMOL “align” command was employed with default parameters, which iteratively optimizes the superposition of two structures by minimizing the RMSD of matched Cα atoms.

For HAstV1 and VA1, the alignment was conducted between their respective AlphaFold2-predicted models and experimentally determined crystal structures (HAstV1 PDB ID: 5EWO and VA1 PDB ID: 8UFO). In the case of MLB2, due to the absence of an experimentally resolved MLB2 spike structure, its AlphaFold2-predicted model was aligned with the closely related MLB1 crystal structure (PDB ID: 7UZT) as a homologous reference. Structural matches were evaluated quantitatively using RMSD values calculated over aligned residue pairs. Residue pairs were defined based on sequence identity and structural overlap within conserved beta-strand and alpha-helical regions.

The alignment accuracy was further validated by analyzing local structural features, including secondary structure elements and conserved domain folds. All structural figures were rendered using PyMOL to visualize superimposed models and highlight regions of structural divergence or conservation.

### Plasmid construction

pET-28a-VA1S, pET-28a-MLB2S, and pET-28a-HAstV1S were constructed for prokaryotic expression of astrovirus spikes by molecular cloning. In brief, spikes of VA1 (corresponding amino acids no. 408–682) and MLB2 (corresponding amino acids no. 417–643) were obtained by amplification of the VA1 and MLB2 containing plasmid (13) through PCR. Then, each of the PCR fragments along with the pET-28a vector, which carries a 6 × His tag at both the N-terminal and C-terminal, was all digested with BamHI and XhoI (TAKARA, Japan). The digested fragments were subsequently ligated with the digested pET-28a vector with a T4 ligase (NEB, US). The constructs were then transformed into Stbl3 cells. Similarly, the HAstV1 spike fragment (corresponding amino acids no. 431–644) was obtained by amplification of HAstV1-PAVIC. The HAstV1 spike fragment was recombined with the pET-28a expression vector using CloneExpress Ultra One Step Cloning Kit V2 (Vazyme, CAT#: C116-02, China). The primers used in this study were listed in Table 4.

**TABLE 4:** Primers for constructing recombinant expression plasmids

Astrovirus	Primer	Oligonucleotide (5'→3')
VA1	VA1S-F	GCGGATCCCCGATACCTCCAACAGG
	VA1S-R	GTGCTCGAGTTTTTCCACAGTTGGCAAG
MLB2	MLB2S-F	GCGGATCCTCATATCCAATGACCCCAGA
	MLB2S-R	GTGCTCGAGACTGTAAATGGGTTCCCATG
HAstV1	HastV1S-F	AGCAAATGGGTCGCGGATCCGAGTATAAAGTTGTCCTCACATT
	HastV1S-R	AGTGGTGGTGGTGGTGGTGCTCGAGAATAGATGTCAGCATCTCAAAC

### Protein expression and purification

The recombinant plasmids were transformed into BL21(DE3) competent cells. A single colony for each expression construct was picked and cultured in 5 mL Luria Broth medium containing ampicillin (100 μg/mL) at 37°C overnight. The culture was then expanded by a dilution of overnight culture at 1:100 and continued for culture at 37°C, 220 rpm for about 2.5 hours until the optical density at 600 nm (OD600) reached 0.6. IPTG was then added to a final concentration of 1 mM, and the culture was continued at 18°C for 18 hours more. The bacteria cells were harvested by centrifuging at 4°C, 3,700 × *g* for 10 min. The supernatant was removed by decanting, and the pellet was added with the cell lysis buffer (20 mM Tris-HCl pH 8.0, 500 mM NaCl, 20 mM imidazole, 2 mM MgCl_2_, 0.0125 U/mL benzonase, and 1 mM Phenylmethylsulfonyl fluoride and resuspended in five times the volume of pellet. The cells were lysed using an ultrasonic cell disruptor (Scientz, China) at 30% power (600 W) for 7 seconds, pausing for 10 seconds for a total of 10 min until the solution became clear. The lysates were centrifuged at 10,000 × *g* for 30 min. The supernatant was further mixed with Ni-NTA beads. After binding with the Ni-NTA beads, it was rotated and mixed at 4°C for 2 hours and then loaded onto an affinity chromatography column (Beyotime, China) for free sedimentation. The column was washed with eight-bed volumes of cell lysis buffer. The beads in the column were eluted with two bed volumes of elution buffer (20 mM, 100 mM, 250 mM, and 500 mM imidazole). All purification steps were conducted at 4°C to preserve protein and activity. Finally, a 10 kDa ultrafiltration (Millipore, USA) was used to exchange the elution buffer with Phosphate-Buffered Saline buffer. The protein was quantified by bicinchoninic acid assay. The protein concentration is determined and stored at −80°C until use.

### Western blot

The three astrovirus proteins were denatured by boiling with 2 × SDS-PAGE sample buffer (Invitrogen) and then resolved on a 10% SDS-PAGE gel through electrophoresis at 120 V for 80 min. PAGE gels were wet transferred onto 0.45 μm polyvinylidene difluoride (PVDF) membranes (Millipore, USA) using the WIX gel transfer machine at 300 mA for 1 hour. The PVDF membranes were blocked by incubation with 5% skim milk (Biosharp, NO. BS102, China) in Tris-buffered saline containing 0.1% Tween-20 (TBST) for 1.5 hours at room temperature. The membranes were then incubated with primary anti-6 × His antibodies (0.15 μg/mL, Proteintech, CAT#: 66005-1-Ig, USA) diluted in the same blocking buffer overnight at 4°C. Following three times of 10 min TBST washes, membranes were probed with Horseradish Peroxidase-conjugated goat anti-mouse secondary antibodies (0.05 μg/m, Abbkinee, CAT#: A21010, China) for 1 hour. After five TBST washes (5 min each), target protein bands were developed and visualized via chemiluminescent reagents (3 min) followed by digital capture using a chemiluminescence imager (Bio-Rad, USA).

### Enzyme-linked immunosorbent assay

HAstV1, MLB2, and VA1 spike proteins were coated onto a high protein binding 96-well plate (Jet, NO. FEP101896, China) with a total amount of 12.5 ng at 4°C for 12 hours. The antigen-coated plate was washed three times with 1 × PBST (containing 0.1% Tween 20, pH adjusted to 7.4) and blocked with 5% skim milk (Biosharp, NO. BS102, China) in PBST at 37°C for 1.5 hours. After three additional washes with 1 × PBST, the plate was incubated with 100 μL inactivated human serum (1:400 dilution) at 37°C for 1 hour, followed by five washes with 1 × PBST. Then, incubated with HRP-labeled goat anti-human IgG antibody (1:250, Beyotime, NO. A0201, China) 100 μL/well at 37°C for 1 hour. After washing with 1 × PBST five times, 3,3',5,5'-Tetramethylbenzidine (Beyotime, NO. P0209, China) was added at room temperature in the dark at 100 μL/well for 20 min. Then, the reaction was terminated with 50 μL 2M H_2_SO_4_ (XIHUA, NO. 1103, China), and the OD450 value was read on a microplate reader (BioTek, USA).

### Immunization of mice

On the day of immunization, 50 μL of blood was collected by cheek bleeding in the mouse as a pre-immune control. The prepared HAstV spike protein (50 μL, 2 mg/mL) with a purity greater than 95% was mixed 1:1 with the adjuvant (Advaccine, China), gently blown, and mixed homogeneously by a syringe. The mixture was then injected intramuscularly into 8-week-old Specific Pathogen Free-grade B6/J mice. Mice were immunized three times on days 0, 14, and 28. A 50 μL blood was collected by cheek bleeding to measure the antibody titer in the serum by indirect ELISA on days 24 and 38. Finally, the mice were sacrificed, and sera were collected.

### Statistical analyses

All statistical analyses were performed using R software (version 4.2.3; R Foundation for Statistical Computing) and GraphPad Prism (version 9.5.0; GraphPad Software). Normal distribution was assessed via Shapiro-Wilk tests (α = 0.05) and variance homogeneity via *F*-tests. Continuous variables were summarized as median IQR for non-normal distributions or mean ± SD for normally distributed data. Between-group comparisons utilized Mann-Whitney *U* tests for non-parametric data, while Student’s *t*-tests were applied to parametric data. Categorical variables were expressed as frequencies (%) and analyzed with Pearson’s *χ*² tests (all expected cell frequencies exceeded 5, precluding the need for Fisher’s exact tests).

Seropositivity thresholds were defined as the 97.5th percentile of optical density (OD450) values measured in negative control (GFP-coated) wells. Ordinal logistic regression models (adjusted for age, sex, HIV status, and co-exposure to other antigens) assessed predictors of graded antibody responses (negative/weak/strong/super strong positive) for HAstV1, MLB2, and VA1. Proportional odds assumptions were verified via Brant tests, and multicollinearity was examined through VIF < 5. Spearman’s correlation analyzed associations between ART initiation delay and antigen-specific OD450 values. A two-tailed *P* < 0.05 defined statistical significance. All analyses were interpreted without adjustment for multiple comparisons.
